# A scissor-guided single-cell framework defines a macrophage-derived risk score for prognostic and immunotherapy stratification in lung adenocarcinoma

**DOI:** 10.3389/fimmu.2026.1827555

**Published:** 2026-05-14

**Authors:** Yueyue Shi, Linyue Hai, Guoqing Hou, Zhiqiang Wang, Lanju Wang, Zhenyu Lv, Xinjian Hao, Shiran Chen, Rong Xia, Junzheng Cao, Shujun Shao

**Affiliations:** 1 Department of Blood Transfusion,The Affiliated Cancer Hospital of Zhengzhou University & Henan Cancer Hospital, Zhengzhou, China; 2 The First Department of Breast Cancer, Tianjin Medical University Cancer Institute and Hospital, National Clinical Research Center for Cancer, Tianjin, China; 3 Zhengzhou University People’s Hospital, Henan Provincial People’s Hospital, Zhengzhou, China; 4 Department of Clinical Laboratory of Shangqiu Second People’s Hospital, Shangqiu, Henan, China; 5 Department of Blood Transfusion, Huashan Hospital, Fudan University, Shanghai, China; 6 Department of Neurosurgery, The Second Affiliated Hospital of Zhengzhou University, Zhengzhou, Henan, China

**Keywords:** LUAD, machine learning, macrophage, SCISSOR, ScRNA-seq, TIMP1

## Abstract

**Background:**

Macrophages are key regulators within the lung adenocarcinoma (LUAD) tumor microenvironment, and their functional states and interaction patterns are closely linked to prognosis and immunotherapy response. However, macrophage-associated risk models derived from single-cell features remain limited.

**Methods:**

Single-cell datasets from GEO were integrated with TCGA-LUAD transcriptomic profiles to characterize macrophage subpopulations. Scissor was applied to map clinical phenotypes onto single cells and identify phenotype-associated macrophage subsets. Prognostic genes were used to construct a machine learning model termed the Scissor-Associated Macrophage Risk Score (SAMRS), which was evaluated across multiple cohorts. Genomic features, immunotherapy response indicators, cell–cell communication, virtual gene knockout, spatial transcriptomics, and functional experiments were further incorporated for mechanistic validation.

**Results:**

Multiple functionally distinct macrophage subsets were identified, and Scissor+ macrophages were associated with poor prognosis and oncogenic pathway activity. Communication analysis placed macrophages at central signaling positions. The SAMRS model achieved robust prognostic stratification across cohorts. High SAMRS scores were linked to higher mutation burden but stronger immune evasion signals, whereas low SAMRS scores showed more favorable predicted immunotherapy response. The model gene TIMP1 demonstrated consistent signals across bulk, single-cell, and spatial analyses, and functional experiments supported its tumor-promoting role.

**Conclusion:**

A single-cell phenotype–guided macrophage risk score, SAMRS, was developed for prognostic stratification and immunotherapy response assessment in LUAD. TIMP1-related regulatory programs may contribute to macrophage-associated tumor progression.

## 1 Introduction

LUAD remains one of the leading causes of cancer-related mortality worldwide despite advances in early detection and systemic therapy (1, 2). Increasing evidence indicates that tumor progression and therapeutic response are not determined solely by malignant epithelial cells but are strongly influenced by the tumor microenvironment (3, 4). Among stromal and immune components, macrophages represent one of the most abundant and functionally versatile cell populations, participating in antigen presentation, inflammatory regulation, matrix remodeling, and immune modulation (5). Their phenotypic plasticity enables dynamic adaptation to local tumor signals, leading to heterogeneous functional states with distinct biological and clinical implications (6).

Recent transcriptomic studies have shown that macrophage-associated gene programs are closely linked to tumor aggressiveness, immune escape, and treatment resistance across multiple cancer types (7). However, most existing macrophage-related signatures are derived from bulk transcriptome data and therefore lack cellular resolution. Bulk-level models cannot distinguish whether prognostic signals originate from specific macrophage subsets or reflect mixed cellular contributions (8). With the development of single-cell RNA sequencing, it has become possible to dissect macrophage heterogeneity at high resolution, revealing multiple transcriptionally and functionally distinct subpopulations within the same tumor (9–11). Translating these single-cell–defined macrophage states into clinically applicable prognostic frameworks remains an important but underexplored task.

Another challenge is how to directly connect single-cell phenotypes with patient-level clinical outcomes. Conventional single-cell clustering identifies transcriptional diversity but does not inherently indicate which cell subsets are associated with prognosis (12). Phenotype-to-cell mapping strategies, such as Scissor, provide a solution by linking bulk clinical traits with single-cell profiles and identifying phenotype-associated cell populations (13). This framework enables the extraction of clinically relevant cellular programs and supports downstream model construction based on biologically grounded cell subsets rather than purely statistical gene selection.

In addition, macrophage-associated signaling is highly dependent on intercellular communication and spatial context (14). Ligand–receptor interactions, regulatory network structure, and tissue spatial organization together shape macrophage functional outputs. Integrating cell–cell communication inference, regulatory network perturbation, and spatial transcriptomics can therefore provide complementary layers of evidence to support candidate gene and pathway selection (15).

In this study, single-cell and bulk transcriptomic data were integratively analyzed to characterize macrophage heterogeneity in LUAD and to identify phenotype-associated macrophage subsets. Based on Scissor-derived macrophage-associated genes, a machine learning prognostic model termed the Scissor-Associated Macrophage Risk Score (SAMRS) was developed and validated across multiple cohorts. Genomic alteration features, immunotherapy response indicators, communication networks, virtual gene perturbation, and spatial transcriptomic patterns were further incorporated to interpret the biological basis of the model. Functional experiments were performed to validate the role of a key model gene, TIMP1. This integrative framework links single-cell macrophage phenotypes with prognostic modeling and functional validation in LUAD.

## 2 Methods

### 2.1 Data sources and public resource acquisition

Single-cell RNA sequencing datasets were obtained from the Gene Expression Omnibus (GEO) repository, including GSE131907 (16) and GSE189357 (17). Raw or processed expression matrices and corresponding metadata were downloaded according to the platform-specific formats provided by GEO. Bulk RNA-seq transcriptomic profiles and matched clinical annotations for lung adenocarcinoma were retrieved from The Cancer Genome Atlas (TCGA) data portal. Independent external validation cohorts were collected from the GEO database, and the specific accession numbers are summarized in the Results section. Functional annotation resources for pathway and gene set enrichment analyses were derived from the Gene Ontology (GO) database and the Kyoto Encyclopedia of Genes and Genomes (KEGG) knowledgebase. Gene set definitions and pathway mappings were obtained from their official annotation repositories. Immunotherapy response prediction metrics were collected from publicly available computational frameworks, including the Tumor Immune Dysfunction and Exclusion (TIDE) (18) platform and the Immunophenoscore (IPS) (19) resource from The Cancer Immunome Atlas (TCIA). Estimates of tumor immune cell infiltration were obtained from the TIMER2.0 (Tumor Immune Estimation Resource, version 2) database (20), which integrates multiple deconvolution algorithms across TCGA cohorts. Pan-cancer gene expression and survival analysis resources were accessed through the GEPIA3.0 web server (21), which provides harmonized analyses based on TCGA and GTEx datasets.

All datasets used in this study were generated by publicly available consortia, and therefore did not require additional institutional ethical approval.

### 2.2 Single-cell RNA sequencing preprocessing, integration, and clustering analysis

Raw single-cell gene expression matrices were processed using the Seurat (22) package (v4.4) in R. Quality control was performed at the cell level to exclude low-quality cells and potential doublets. Specifically, cells were retained only if they met the following criteria: the number of detected genes (nFeature_RNA) ranged from 500 to 10,000, the total UMI count (nCount_RNA) ranged from 1,000 to 100,000, the percentage of mitochondrial transcripts (percent.mt) was no more than 20%, and the percentage of hemoglobin-related transcripts (percent.HB) was no more than 5%. Genes expressed in only a very small fraction of cells were also excluded to reduce background noise. The percentage of mitochondrial transcripts per cell was calculated based on mitochondrial gene sets and incorporated into filtering criteria. After filtering, expression values were normalized using a global-scaling strategy and log-transformed. Highly variable genes were identified from the normalized matrix and used for downstream dimensionality reduction. Principal component analysis (PCA) was conducted to capture major sources of transcriptional variation. To minimize technical heterogeneity across samples, batch effects were corrected using the Harmony algorithm applied to the PCA embeddings, with sample identity (orig.ident) specified as the correction variable. Harmony integration was performed using the RunHarmony() function with group.by.vars = “orig.ident” and plot_convergence = TRUE. For downstream analysis, the first 20 Harmony dimensions were used for neighbor graph construction and UMAP visualization (23). The Harmony-adjusted low-dimensional space was then used to build a shared nearest-neighbor graph. Graph-based clustering was subsequently performed to identify transcriptionally coherent cell populations, with cluster resolution tuned according to dataset complexity. For visualization, Uniform Manifold Approximation and Projection (UMAP) was generated based on the Harmony-corrected components. Cluster annotation was determined through combined evaluation of canonical marker genes and cluster-specific differentially expressed genes identified using Seurat’s marker detection framework. These annotated clusters were used for all downstream cell-type–level and functional analyses.

### 2.3 Inference of intercellular signaling networks with CellChat

Intercellular communication patterns were inferred using the CellChat (24) framework in R. Normalized single-cell expression data and curated cell-type annotations derived from Seurat clustering were used as input. A CellChat object was constructed from the expression matrix together with cell identity labels, and genes were mapped to the built-in ligand–receptor interaction database provided by CellChat. Genes with low detection rates across cells were excluded based on internal filtering criteria. Overexpressed ligands and receptors were identified for each cell group, followed by computation of potential interaction probabilities between sender and receiver populations using the CellChat probabilistic model. Communication probabilities were aggregated at the signaling pathway level to quantify pathway-specific interaction strengths. The inferred communication network was subsequently analyzed at both global and pathway-resolved scales. Network centrality measures were computed to characterize dominant signaling senders, receivers, mediators, and influencers among cell populations. Comparative analyses of signaling strength and interaction number across cell groups were performed using the built-in network analysis functions. Selected signaling pathways were further visualized using circle plots, hierarchy plots, and bubble diagrams to illustrate ligand–receptor contributions and directional communication structure.

### 2.4 Transcription factor regulatory network analysis using SCENIC

Regulatory network inference at the single-cell level was performed using the SCENIC (25) workflow. Normalized single-cell expression data were used to construct gene co-expression modules and identify candidate transcription factor–target relationships. Putative regulons were defined by integrating co-expression with motif enrichment analysis to retain transcription factor–binding support. Regulon activity scores were subsequently quantified in individual cells using AUCell, generating a regulon activity matrix. These activity profiles were used to compare transcription factor regulatory programs across cell populations and to characterize cell state–associated regulatory patterns.

### 2.5 Pseudotime trajectory inference using slingshot

Cellular trajectory and pseudotime ordering were inferred using the Slingshot algorithm (26). Dimension-reduced embeddings and cluster labels derived from Seurat analysis were used as inputs. Slingshot fits lineage curves across clusters in a low-dimensional space and reconstructs potential developmental trajectories without requiring predefined paths. Lineage structures were estimated by connecting transcriptionally related clusters, followed by pseudotime assignment for individual cells along each inferred lineage. The resulting pseudotime values were used to characterize dynamic transcriptional changes and state transitions across cell populations.

### 2.6 Phenotype-associated cell subset identification using scissor

Phenotype–cell association analysis was conducted using the Scissor algorithm to link single-cell profiles with bulk-level clinical outcomes (13). Normalized single-cell expression matrices and matched bulk transcriptomic data with survival or phenotype annotations were used as joint inputs. Feature alignment between single-cell and bulk datasets was performed based on shared gene sets prior to modeling. Scissor applies a regression-based framework to identify cells whose transcriptional patterns are positively or negatively associated with the bulk phenotype of interest. Cells were classified into phenotype-associated (Scissor+) and phenotype-opposed (Scissor−) subsets according to model selection results. Downstream differential expression and functional analyses were performed to characterize molecular features enriched in phenotype-associated cell populations.

### 2.7 In silico gene knockout analysis using scTenifoldKnk

Virtual gene perturbation analysis was performed using the scTenifoldKnk (27) framework to estimate transcriptional network responses to single-gene knockout at the single-cell level. Normalized single-cell expression data were used to construct gene regulatory networks based on cell-level transcriptional covariance structure. For each candidate gene, a virtual knockout was simulated by computationally removing its regulatory influence within the inferred network model. The perturbed network was then compared with the original network to quantify downstream transcriptional shifts and network rewiring effects. Genes showing the largest predicted regulatory displacement were considered putative downstream targets of the perturbed gene.

The resulting perturbation impact profiles were used to characterize gene-centered regulatory dependencies and to prioritize genes with broad network-level influence.

### 2.8 Prognostic evaluation of cell population signatures

Cluster-specific marker genes were identified from single-cell data by differential expression analysis comparing each cell population against all remaining cells. Markers were ranked by effect size and statistical significance, and the top-ranked genes for each cluster were selected to construct cell population–specific gene signatures.To evaluate the prognostic relevance of these cell population signatures at the cohort level, bulk RNA-seq data and corresponding survival information from the TCGA lung adenocarcinoma dataset were used. For each sample, enrichment scores of cluster-derived gene signatures were quantified using single-sample gene set enrichment analysis (ssGSEA) (28), generating a per-sample activity score representing the relative abundance or activation of each cell population–associated program. Patients were subsequently stratified into high-score and low-score groups based on the optimal cutoff value determined by outcome-oriented threshold selection. Kaplan–Meier survival analysis and log-rank testing were then performed to compare overall survival between groups. Hazard ratios and confidence intervals were estimated using Cox proportional hazards models where applicable.

### 2.9 Machine learning–based prognostic model construction and evaluation

Candidate prognostic features were first screened using survival association analyses in the training cohort. The TCGA-LUAD cohort was used as the training set for feature screening and model construction, whereas the independent GEO cohorts were used as external validation sets to assess the generalizability and robustness of the model. Multiple machine learning algorithms were incorporated for prognostic model construction, including Elastic Net, LASSO, Ridge, Stepwise Cox, survivalSVM, CoxBoost, SuperPC, plsRcox, random survival forest (RSF), and gradient boosting machine (GBM). Candidate models were trained in the training cohort and subsequently evaluated in independent validation cohorts. Model selection was primarily based on concordance index (C-index) performance and cross-cohort predictive stability, and the best-performing model was retained as the final prognostic model.

A risk score for each patient was calculated according to the final model formula derived from the selected features and their coefficients. Patients were stratified into high-risk and low-risk groups using the median risk score as the cutoff. Survival differences between risk groups were assessed using Kaplan–Meier analysis and log-rank testing, and effect sizes were estimated using Cox proportional hazards regression. Time-dependent receiver operating characteristic (ROC) curves were generated to quantify predictive accuracy at different follow-up time points. To facilitate clinical application, a prognostic nomogram integrating the model-derived risk score and available clinical variables was established. Calibration analysis and discrimination metrics were used to evaluate nomogram performance.

### 2.10 Genomic alteration landscape characterization

Somatic mutation data for lung adenocarcinoma were obtained from TCGA through the Genomic Data Commons portal. Masked somatic mutation files were downloaded and consolidated into a unified mutation annotation format for downstream analysis. Samples without matched clinical or risk stratification information were excluded prior to modeling.

Mutation profiling and visualization were performed using a dedicated mutation analysis framework. The overall mutation burden, variant classification distribution, and mutation spectra were summarized at the cohort level. Frequently altered genes were identified based on mutation frequency and displayed using waterfall-style mutation maps. To explore associations between genomic alterations and clinical characteristics, mutation patterns were compared across predefined clinical and risk subgroups. Selected model-related genes were further examined for mutation status distribution across samples. Pairwise mutation co-occurrence and mutual exclusivity relationships among top mutated genes were evaluated using statistical interaction analysis and visualized as gene–gene interaction networks.

### 2.11 Immunotherapy response–related metric evaluation

To estimate potential immunotherapy benefit across risk subgroups, multiple immune response–associated indicators were systematically evaluated. Tumor mutational burden (TMB) was calculated from somatic mutation profiles by quantifying the number of nonsynonymous mutations per sample, and TMB levels were compared between predefined groups. IPS, which reflects tumor immunogenicity based on immune-related gene expression patterns, was obtained from a publicly available cancer immunome resource. IPS values were used to assess differences in predicted immune checkpoint blockade responsiveness between subgroups. TIDE scores were derived from the TIDE computational framework to model mechanisms of T-cell dysfunction and immune exclusion. Higher TIDE scores indicate increased likelihood of immune escape and reduced response to checkpoint blockade. Comparative analyses of TIDE metrics were performed across groups to infer relative immunotherapy sensitivity.

These complementary indicators were integrated to provide a multi-dimensional evaluation of predicted immunotherapy response potential.

### 2.12 Spatial transcriptomics preprocessing and RCTD-based cell type deconvolution

Spatial transcriptomics data were processed using Seurat (v5.2.1). Low-information features and spots were filtered by removing genes with very low total read counts and spatial barcodes with insufficient transcript coverage. High-resolution tissue images provided with the datasets were used for spatial alignment and visualization. Expression matrices were normalized using the SCTransform framework to stabilize variance across spots. Cell type decomposition of spatial spots was performed using the RCTD algorithm implemented in the spacexr package (v2.2.1). A single-cell reference atlas generated from the preprocessed scRNA-seq dataset was used to construct the deconvolution reference, incorporating a curated panel of representative cell types. Total UMI counts per cell were included as scaling factors when building the reference model. For each spatial sample, count matrices, spot-level UMI totals, and spatial coordinates were assembled into a spatial RNA object prior to model fitting. Deconvolution was executed under the full modeling mode with default parameter settings for remaining options.

### 2.13 Spatial co-localization analysis based on Ripley’s K function

To quantify spatial association between selected gene-positive niches, spatial co-localization analysis was conducted using a point pattern framework. Gene expression values derived from variance-stabilized spatial data were thresholded to define marker-positive spots for each gene of interest. Samples with insufficient numbers of positive spots in either category were excluded to ensure statistical stability. Coordinates of marker-positive spots were converted into multi-type spatial point patterns. Cross-type Ripley’s K and L functions were computed across a continuous distance range to evaluate deviation from spatial independence between spot categories. Statistical significance was assessed using Monte Carlo simulation to generate confidence envelopes under the null hypothesis of random spatial distribution. Effect magnitude was summarized using distance-dependent deviation metrics and aggregation indices derived from the transformed L-function curves. Spatial distances were scaled to physical units according to image resolution metadata.

### 2.14 Cell culture

Human lung cancer cell lines A549 and H1299 were maintained under standard conditions. Cells were cultured in complete medium supplemented with fetal bovine serum and routinely incubated at 37 °C in a humidified atmosphere containing 5% CO_2_. Cells in logarithmic growth phase were used for subsequent experiments, and cultures were periodically examined to exclude mycoplasma contamination.

### 2.15 siRNA-mediated TIMP1 knockdown

Transient silencing of TIMP1 was achieved using small interfering RNAs (siRNAs) together with a non-targeting control siRNA. Cells were plated one day prior to transfection to reach appropriate confluence and transfected with siRNA using a lipid-based transfection reagent according to the manufacturer’s protocol.

To reduce sequence-dependent off-target effects, two independent TIMP1-targeting siRNAs were applied. The sense strand sequences (5′–3′) were:

TIMP1 siRNA-A (5′–3′): CCCCACCTTATACCAGCGTTATGAGAT.

TIMP1 siRNA-B (5′–3′): GATCAAGATGACCAAGATGTATT.

Cells were collected at designated time points after transfection for downstream molecular and functional analyses. Knockdown efficiency was verified at the RNA level by quantitative RT–PCR before subsequent experiments were performed.

### 2.16 Quantitative RT–PCR

Total RNA was isolated from cultured cells using a standard RNA extraction procedure according to the manufacturer’s protocol. RNA concentration and purity were evaluated prior to downstream processing. Complementary DNA (cDNA) was synthesized from equal amounts of total RNA using a reverse transcription system under recommended reaction conditions. Quantitative PCR was carried out using a SYBR Green–based detection chemistry on a real-time PCR platform. Each sample was analyzed with technical replicates, and negative controls without template were included. Relative transcript levels were calculated using the 2^−ΔΔCt method with a housekeeping gene as the internal reference.

The primer sequences used for TIMP1 amplification were:

TIMP1 forward (5′–3′): ATTCCGACCTCGTCATCAGG.

TIMP1 reverse (5′–3′): GGACCTGTGGAAGTATCCGC.

### 2.17 CCK-8 cell viability assay

Cell viability was evaluated using a colorimetric metabolic activity assay based on tetrazolium salt conversion. Transfected and control cells were seeded into 96-well plates at equal densities and cultured for indicated time periods. At each time point, CCK-8 working solution was added to each well and incubated under standard culture conditions. Absorbance was measured at 450 nm using a microplate reader. Background wells containing medium without cells were included for correction.

### 2.18 EdU incorporation assay

Cell proliferative activity was measured using a nucleoside analog incorporation assay. Cells were incubated with EdU labeling reagent for a defined period under standard culture conditions. After labeling, cells were fixed, permeabilized, and subjected to click-reaction staining according to the kit protocol. Nuclei were counterstained, and labeled cells were visualized using fluorescence microscopy. The proportion of EdU-positive cells was quantified from multiple random fields.

### 2.19 Transwell migration and invasion assays

Cell migratory and invasive capacities were assessed using Transwell chamber systems. For migration assays, cells suspended in serum-free medium were added to the upper chamber, while medium containing chemoattractant was placed in the lower chamber. After incubation, non-migrated cells on the upper surface were removed, and migrated cells on the lower membrane surface were fixed and stained for counting.For invasion assays, the upper chamber membrane was pre-coated with extracellular matrix gel prior to cell seeding. The remaining procedures were performed similarly to the migration assay. Cells were counted in multiple microscopic fields.

### 2.20 Wound-healing assay

Cell migration was further evaluated using a wound-closure assay. Cells were seeded into culture plates and grown to near confluence. A linear scratch was generated across the cell monolayer using a sterile pipette tip. Detached cells were removed by washing, and fresh medium was added. Images were captured at defined time points using phase-contrast microscopy, and wound closure was assessed by measuring the change in scratch width.

### 2.21 Statistical analysis

All statistical analyses were conducted using R software and GraphPad Prism unless otherwise specified. Continuous variables between two groups were compared using either Student’s t-test or the Wilcoxon rank-sum test according to data distribution characteristics. Comparisons among multiple groups were performed using analysis of variance or nonparametric alternatives when appropriate. Survival analyses were carried out using Kaplan–Meier methods with log-rank tests. Cox proportional hazards regression models were applied for univariate and multivariate survival evaluation. Optimal cutoff values for risk stratification were determined using data-driven approaches implemented in survival analysis packages. Correlation analyses were performed using Spearman or Pearson methods depending on variable type and distribution. For functional enrichment and pathway score comparisons, groupwise statistical testing was performed using rank-based or parametric tests as appropriate. Cellular and animal experimental data were derived from independent biological replicates and presented as mean ± standard deviation unless otherwise stated. A two-sided P value less than 0.05 was considered statistically significant.

## 3 Results

### 3.1 System-level single-cell integration delineates cellular composition patterns in LUAD

**Figure 1:**
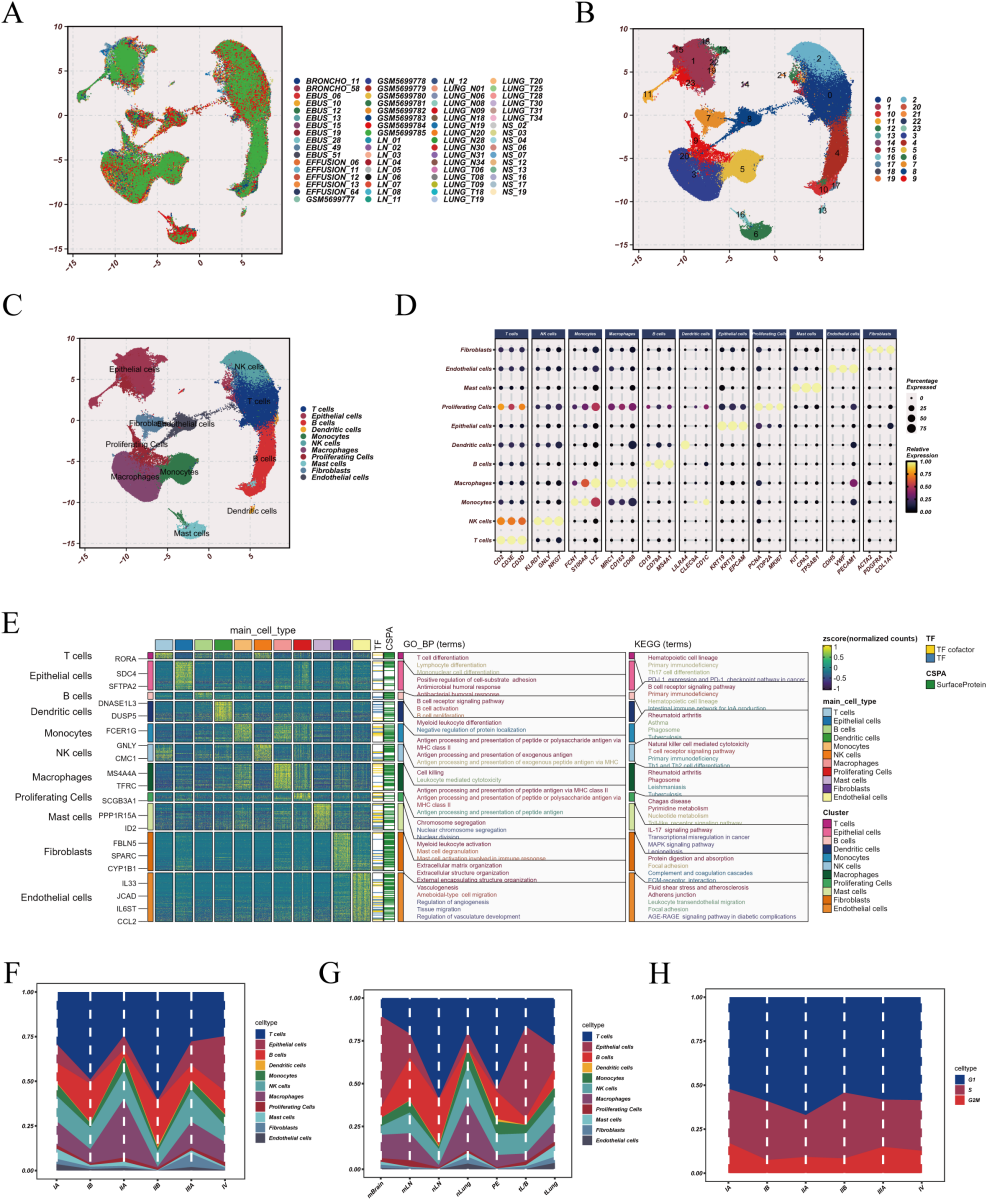
Single-cell transcriptomic landscape and cell-type characterization. **(A)** UMAP visualization showing the distribution of cells across individual samples after quality control and integration. Each color represents one sample source. **(B)** Unsupervised clustering results displayed on the UMAP embedding, with distinct clusters identified based on transcriptional similarity. **(C)** Annotated cell populations projected onto the UMAP space according to canonical marker gene expression patterns. **(D)** Dot plot illustrating representative marker genes across major cell populations, with dot size indicating the proportion of expressing cells and color intensity representing relative expression level. **(E)** Functional enrichment analysis of cluster-specific marker genes, showing significantly enriched Gene Ontology (GO) and KEGG pathway terms for major cell types. **(F)** Relative cell-type composition across clinical stage subgroups, showing variation in macrophage proportions. **(G)** Comparison of macrophage abundance across normal lung tissue, metastatic tumors, advanced tumors, and early-stage lung adenocarcinoma samples. **(H)** Cell-cycle state distribution across different clinical stages.

After dataset integration, Harmony-based correction effectively mitigated inter-sample batch effects, resulting in well-aligned cell distributions across samples in the low-dimensional embedding (**Figure 1A**). Graph-based clustering resolved the integrated dataset into 24 transcriptionally distinct clusters (**Figure 1B**). Cluster annotation based on canonical marker genes grouped these clusters into 11 major cell populations, including macrophages, monocytes, epithelial cells, mast cells, dendritic cells, lymphocyte subsets, and stromal populations (**Figure 1C**). Marker gene visualization demonstrated strong population-restricted expression patterns, supporting the specificity of cell identity assignments (**Figure 1D**). Functional enrichment analysis of macrophage-enriched marker genes showed predominant enrichment in antigen processing and antigen presentation programs, particularly pathways related to peptide antigen presentation via MHC class II and related immune regulatory processes (**Figure 1E**), consistent with an antigen-presenting functional phenotype. Cell composition analysis across clinical stage subgroups revealed increased macrophage proportions in stage IIA and stage IIIA samples relative to other stages **Figure 1F**), suggesting stage-associated remodeling of the myeloid compartment. When compared across tissue contexts, macrophage abundance was higher in normal lung tissue than in early-stage LUAD, metastatic lesions, or more advanced tumors (**Figure 1G**), indicating that macrophage enrichment may represent an early microenvironmental feature rather than a late-stage accumulation pattern. In contrast, comparison of inferred cell-cycle states across clinical stages did not show substantial shifts in overall proliferation state distributions (**Figure 1H**), indicating that stage-associated differences were more strongly reflected in immune composition than in global cell-cycle activity.

### 3.2 Subclustering of macrophages reveals transcriptionally and functionally distinct states

**Figure 2:**
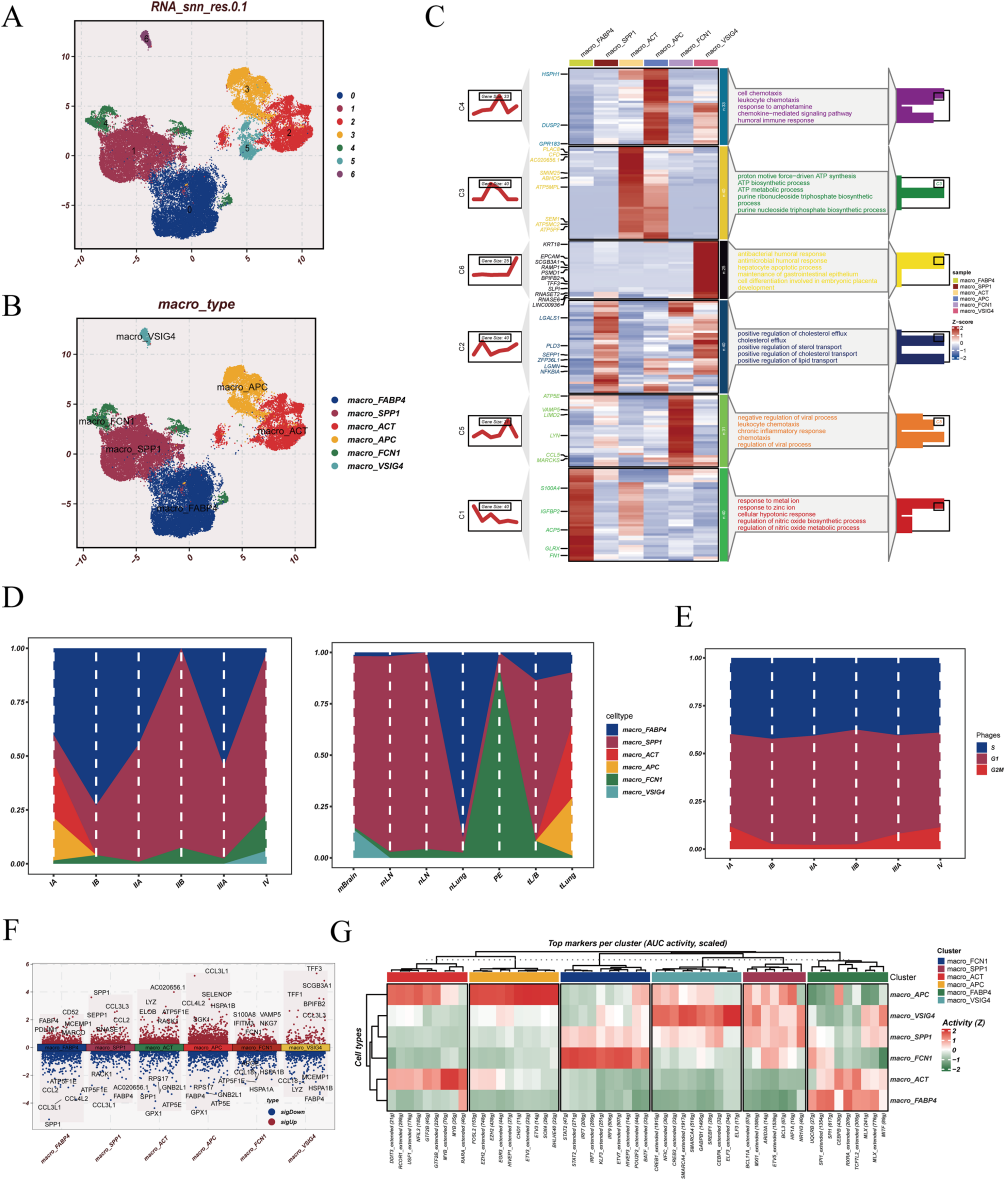
Macrophage subclustering and regulatory characterization. **(A)** UMAP projection of reclustered macrophages showing distinct macrophage subpopulations. **(B)** Annotation of macrophage subsets based on subtype-specific marker gene expression. **(C)** Heatmap of representative marker genes across macrophage subsets with corresponding functional enrichment results. **(D)** Relative abundance of macrophage subsets across clinical stage groups. **(E)** Distribution of inferred cell-cycle states among macrophage subsets. **(F)** Differentially expressed genes across macrophage subsets. **(G)** Inferred transcription factor activity across macrophage subsets based on regulon analysis.

Macrophage-lineage cells were further isolated and reclustered, resolving six transcriptionally distinct macrophage subsets, annotated as macro_FABP4, macro_SPP1, macro_ACT, macro_APC, macro_FCN1, and macro_VSIG4 based on subtype-defining marker genes (**Figures 2A, B**). Functional profiling of subset-specific marker programs revealed divergent biological orientations across macrophage states (**Figure 2C**). The macro_FABP4 subset was primarily associated with leukocyte migration and chemokine-driven signaling activity, indicating a chemotactic and immune recruitment–related phenotype. The macro_SPP1 subset showed dominant enrichment in mitochondrial energy production and nucleotide triphosphate biosynthesis pathways, consistent with a metabolically active state. The macro_ACT subset was linked to humoral antimicrobial responses and epithelial-supportive processes, suggesting host-defense and tissue-interactive functions. The macro_APC subset was characterized by lipid handling and cholesterol transport–related programs, indicating involvement in sterol trafficking and metabolic regulation. The macro_FCN1 subset was enriched for inflammatory and antiviral regulatory processes, reflecting immune-reactive characteristics. The macro_VSIG4 subset showed signatures related to metal ion response and nitric oxide regulation, consistent with immunomodulatory and stress-response features. Stage- and context-stratified composition analysis demonstrated non-uniform distribution of macrophage subsets (**Figure 2D**). We further displayed the cell-cycle distribution across different tissue origins in **Figure 2E**, and no obvious differences were observed. **Figure 2F** shows the differentially expressed genes among distinct macrophage subtypes. The macro_FCN1 subset was markedly enriched in pleural effusion–associated metastatic LUAD samples, whereas macro_SPP1 cells were scarce in normal lung tissue but consistently expanded across tumor samples. These patterns indicate subset-selective redistribution across tissue contexts rather than uniform macrophage expansion.

Regulon-based transcription factor activity inference further showed distinct upstream regulatory programs across macrophage subsets, supporting the presence of state-specific regulatory control architectures (**Figure 2G**).

### 3.3 Phenotype-linked macrophage states identified by Scissor show distinct functional and prognostic associations

**Figure 3:**
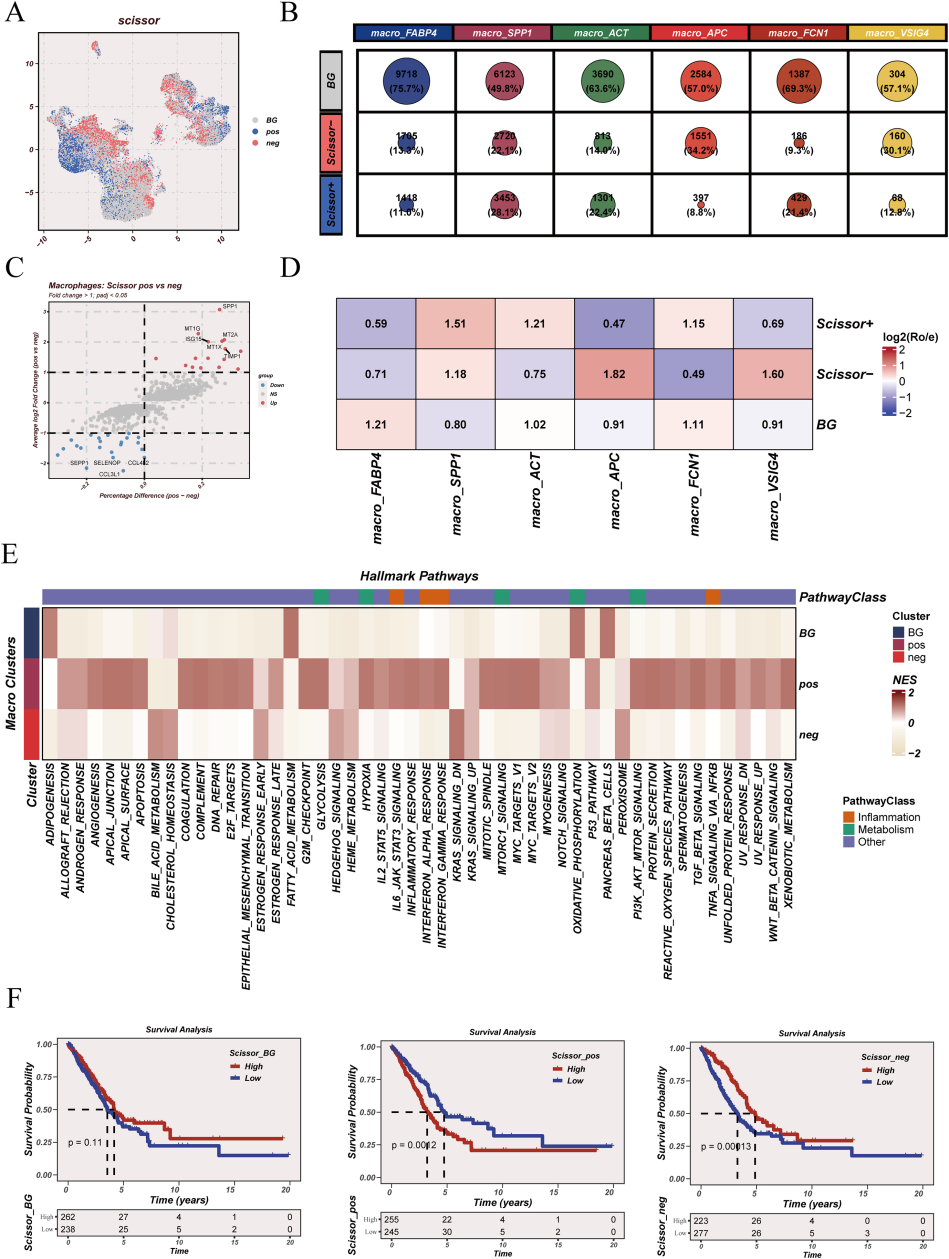
Scissor-based identification of phenotype-associated macrophage subsets and their clinical relevance. **(A)** UMAP projection of macrophages colored by Scissor classification, showing phenotype-associated (Scissor+), phenotype-opposed (Scissor−), and background cells. **(B)** Distribution and abundance of macrophage subsets across Scissor+ and Scissor− groups. **(C)** Differential gene expression landscape between Scissor+ and Scissor− macrophages displayed as a volcano plot. **(D)** Ratio of observed-to-expected (Ro/e) enrichment of macrophage subsets across Scissor-defined groups. **(E)** Hallmark pathway enrichment profiles across Scissor+ and Scissor− macrophage populations. **(F)** Kaplan–Meier survival curves comparing high and low groups defined by Scissor-derived signatures.

Scissor analysis was used to link macrophage single-cell states with bulk-level clinical phenotypes, classifying macrophages into Scissor+, Scissor−, and background (BG) groups (**Figure 3A**). Subset-level composition revealed an uneven distribution of Scissor labels across macrophage states (**Figure 3B**). Notably, the macro_SPP1 subset contained the highest fraction of Scissor+ cells, whereas macro_FABP4 exhibited the lowest Scissor+ representation, indicating subset-specific phenotype linkage rather than a uniform macrophage effect. Differentially expressed genes between Scissor+ and Scissor− macrophages were summarized using a volcano plot to visualize the global expression shift associated with Scissor classification (**Figure 3C**). Observed-to-expected (Ro/e) enrichment analysis further quantified subset bias across Scissor-defined groups (**Figure 3D**). Consistent with the composition patterns, macro_SPP1 showed preferential enrichment within Scissor+ cells, while macro_FABP4 showed relative depletion in Scissor+. In contrast, the Scissor− group displayed an opposing enrichment pattern across subsets, and BG cells did not show pronounced subset skewing. Hallmark pathway analysis demonstrated that Scissor+ macrophages exhibited stronger associations with tumor-relevant transcriptional programs, including pathways commonly linked to malignant progression and microenvironmental remodeling, whereas Scissor− macrophages showed comparatively weaker pathway coupling (**Figure 3E**). Clinically, survival analysis indicated that Scissor+-associated macrophage signatures stratified patients toward poorer outcomes, while Scissor− signatures were associated with favorable prognosis; the BG group showed no apparent prognostic impact (**Figure 3F**).

### 3.4 Cell–cell communication analysis identifies macrophage-centered FN1 signaling activity

**Figure 4:**
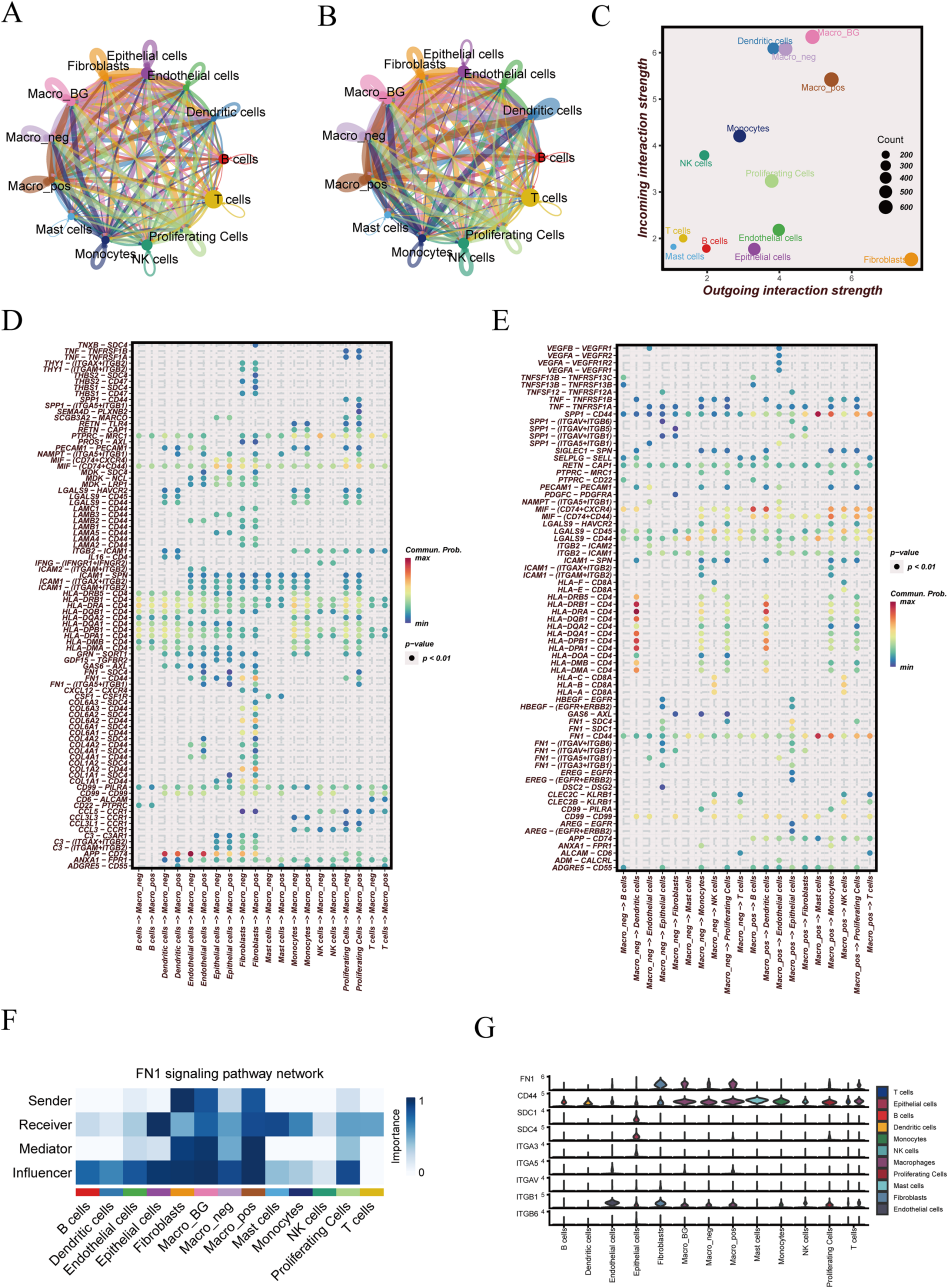
Cell–cell communication network and FN1 signaling analysis. **(A)** Number of inferred interactions among major cell populations. **(B)** Interaction network weighted by communication strength. **(C)** Incoming and outgoing signaling strength across cell populations. **(D)** Communication patterns received by macrophages. **(E)** Communication patterns sent by macrophages. **(F)** Functional role distribution of the FN1 signaling pathway across cell populations. **(G)** Expression patterns of FN1 pathway–related genes in major cell types.

CellChat analysis revealed a densely connected communication network among major cell populations (**Figure 4A**). When interactions were weighted by strength, stromal and myeloid compartments remained central in the network, indicating that high-intensity signaling was not evenly distributed across all cell types (**Figure 4B**). Directionality analysis distinguished dominant senders and receivers (**Figure 4C**). Fibroblasts showed the highest outgoing interaction strength while maintaining relatively low incoming strength, consistent with a “signal-exporting” role. In contrast, dendritic cells and macrophage background cells (Macro_BG) localized at the upper region of the incoming-strength axis, indicating strong signal reception. Monocytes occupied an intermediate position with substantial incoming signaling, whereas T/B cells and mast cells clustered near the low outgoing/incoming region. Focusing on macrophages, receptor-side analysis (macrophages as receivers) showed broad ligand inputs from multiple lineages, with particularly prominent contributions from stromal compartments and immune populations (**Figure 4D**). Conversely, sender-side analysis (macrophages as ligand sources) demonstrated that macrophages also delivered outgoing cues to diverse target cells rather than acting as passive recipients (**Figure 4E**). At the pathway level, FN1 signaling emerged as a key axis in the macrophage-centered communication landscape (**Figure 4F**). The FN1 pathway role matrix indicated that fibroblasts and macrophage subsets contributed strongly as FN1 signal senders and network influencers, whereas macrophage subsets and monocytes showed notable receiver involvement, supporting a distributed FN1 signaling architecture rather than a single-node pathway. Consistently, FN1-related signaling components displayed structured expression across cell types, with higher expression concentrated in stromal/myeloid compartments (**Figure 4G**).

### 3.5 Trajectory-informed macrophage state transitions reveal lineage-specific programs and prognostic relevance

**Figure 5:**
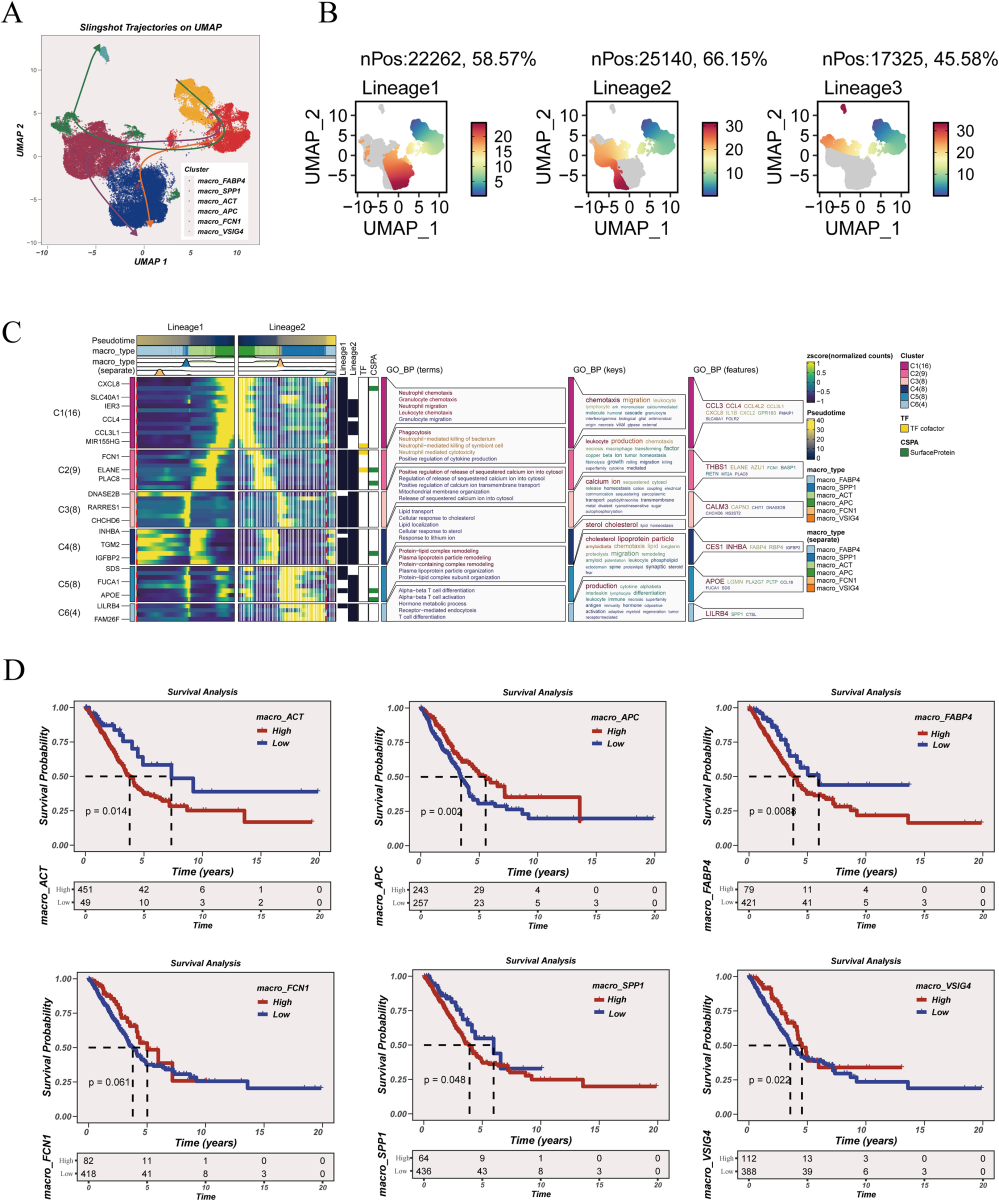
Slingshot-based macrophage trajectories and prognostic relevance of subtype programs. **(A)** Slingshot trajectory inference overlaid on the macrophage UMAP. **(B)** Pseudotime distribution across inferred lineages. **(C)** Dynamic gene expression patterns along macrophage trajectories and functional annotation of associated gene modules. **(D)** Kaplan–Meier survival analysis of macrophage subtype–associated signatures.

Trajectory inference using Slingshot identified multiple macrophage lineage structures overlaid on the macrophage UMAP, indicating that macrophage subsets could be connected by continuous transcriptional transitions rather than forming fully isolated states (**Figure 5A**). Three lineages were resolved, with a substantial fraction of macrophages assigned to each lineage: Lineage 1 (nPos = 22,262; 58.57%), Lineage 2 (nPos = 25,140; 66.15%), and Lineage 3 (nPos = 17,325; 45.58%) (**Figure 5B**). The pseudotime gradients showed lineage-specific spatial patterns across the UMAP manifold, supporting heterogeneity in trajectory usage among macrophage subsets. Pseudotime-ordered expression heatmaps further revealed structured gene program shifts along the inferred trajectories (**Figure 5C**). Distinct gene blocks exhibited coordinated activation or attenuation across pseudotime, and functional annotation of these trajectory-linked modules highlighted differential biological emphases along the lineages, consistent with state-specific immune programs and metabolic remodeling patterns rather than uniform changes. Finally, the clinical relevance of macrophage subtype programs was assessed by survival analysis (**Figure 5D**). High scores for macro_ACT, macro_FABP4, macro_FCN1, and macro_SPP1 were associated with significantly worse overall survival, whereas higher macro_APC and macro_VSIG4 scores were associated with more favorable survival trends. Together, these results connect macrophage trajectory-linked state programs to clinically meaningful outcome stratification.

### 3.6 Machine learning–based modeling of Scissor-associated genes enables prognostic risk stratification

**Figure 6:**
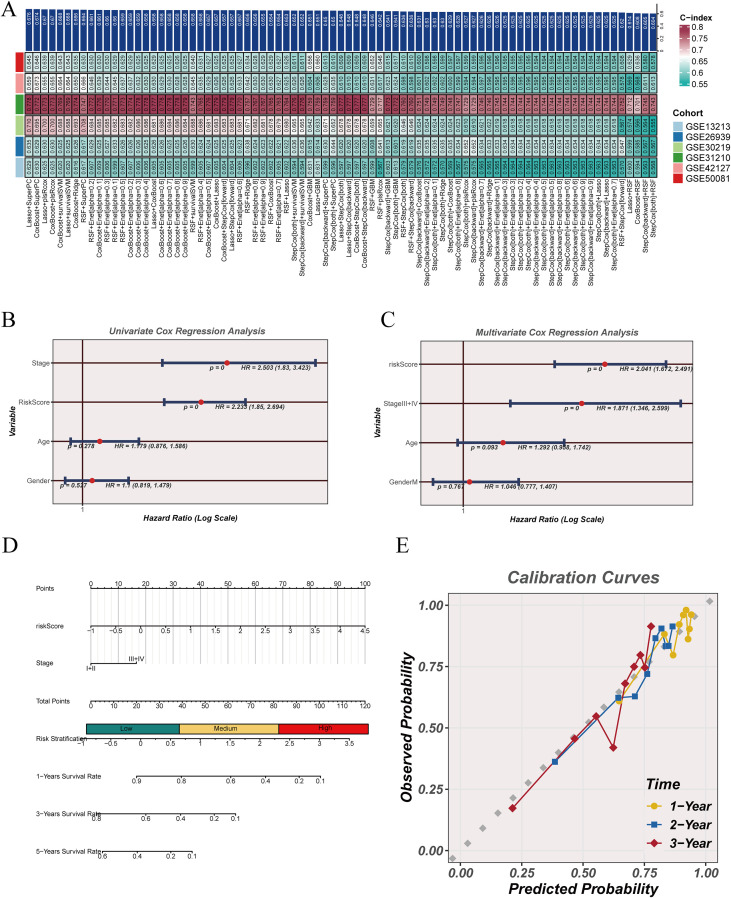
Machine learning–derived prognostic model based on scissor-associated genes. **(A)** Performance comparison of multiple machine learning algorithms across independent cohorts using C-index evaluation. **(B)** Univariate Cox regression analysis of clinical variables and model-derived risk score. **(C)** Multivariate Cox regression analysis assessing the independent prognostic value of the risk score. **(D)** Nomogram integrating risk score with clinical variables for survival prediction. **(E)** Calibration curves evaluating agreement between predicted and observed survival probabilities at different time points.

To convert Scissor-linked macrophage signals into a clinically applicable prognostic framework, marker genes derived from Scissor+ macrophage populations were first screened using univariate Cox regression, and survival-associated candidates were retained for model development (**Supplementary Table 1**). Based on these genes, a machine learning–derived prognostic model, termed the Scissor-Associated Macrophage Risk Score (SAMRS), was constructed. Multiple machine learning algorithms were evaluated for SAMRS model construction, and their predictive performance was compared across independent cohorts using concordance index metrics (**Figure 6A**). Several algorithmic strategies demonstrated consistently higher C-index values across datasets, indicating stable cross-cohort predictive robustness of the SAMRS framework. Clinical association analysis showed that the SAMRS score was significantly associated with overall survival in univariate Cox regression alongside established clinical variables (**Figure 6B**). After adjustment for stage, age, and gender in multivariate Cox models, SAMRS remained statistically significant, supporting its independent prognostic value (**Figure 6C**). To enhance clinical interpretability, a nomogram integrating SAMRS with clinical parameters was established to estimate individual survival probability (**Figure 6D**). Calibration analysis demonstrated close agreement between predicted and observed survival probabilities at 1-, 2-, and 3-year time points (**Figure 6E**), indicating favorable model calibration.

Using the median SAMRS score as the stratification threshold, patients were divided into high- and low-risk groups across the TCGA discovery cohort and multiple external validation cohorts. Survival analysis consistently showed significantly worse outcomes in the SAMRS high-risk group across datasets (**Supplementary Figure 1A**), demonstrating reproducible risk discrimination ability. Time-dependent ROC analysis further supported the predictive performance of SAMRS, with stable AUC values observed for 1-, 3-, and 5-year survival across cohorts (**Supplementary Figure 1B**), indicating sustained temporal predictive accuracy.

To further evaluate the relative performance of SAMRS in the context of existing LUAD prognostic models, we systematically collected 20 previously published LUAD risk signatures and compared their predictive performance with that of SAMRS across the TCGA cohort and multiple independent GEO cohorts. Using the C-index as the primary evaluation metric, SAMRS showed the highest or among the highest predictive performance in most datasets and exhibited superior overall cross-cohort stability compared with previously reported models (**Supplementary Figure 2**). These findings further support the robustness and potential clinical utility of SAMRS for prognostic stratification in LUAD.

### 3.7 Genomic alteration patterns and immunotherapy response indicators associated with SAMRS

**Figure 7:**
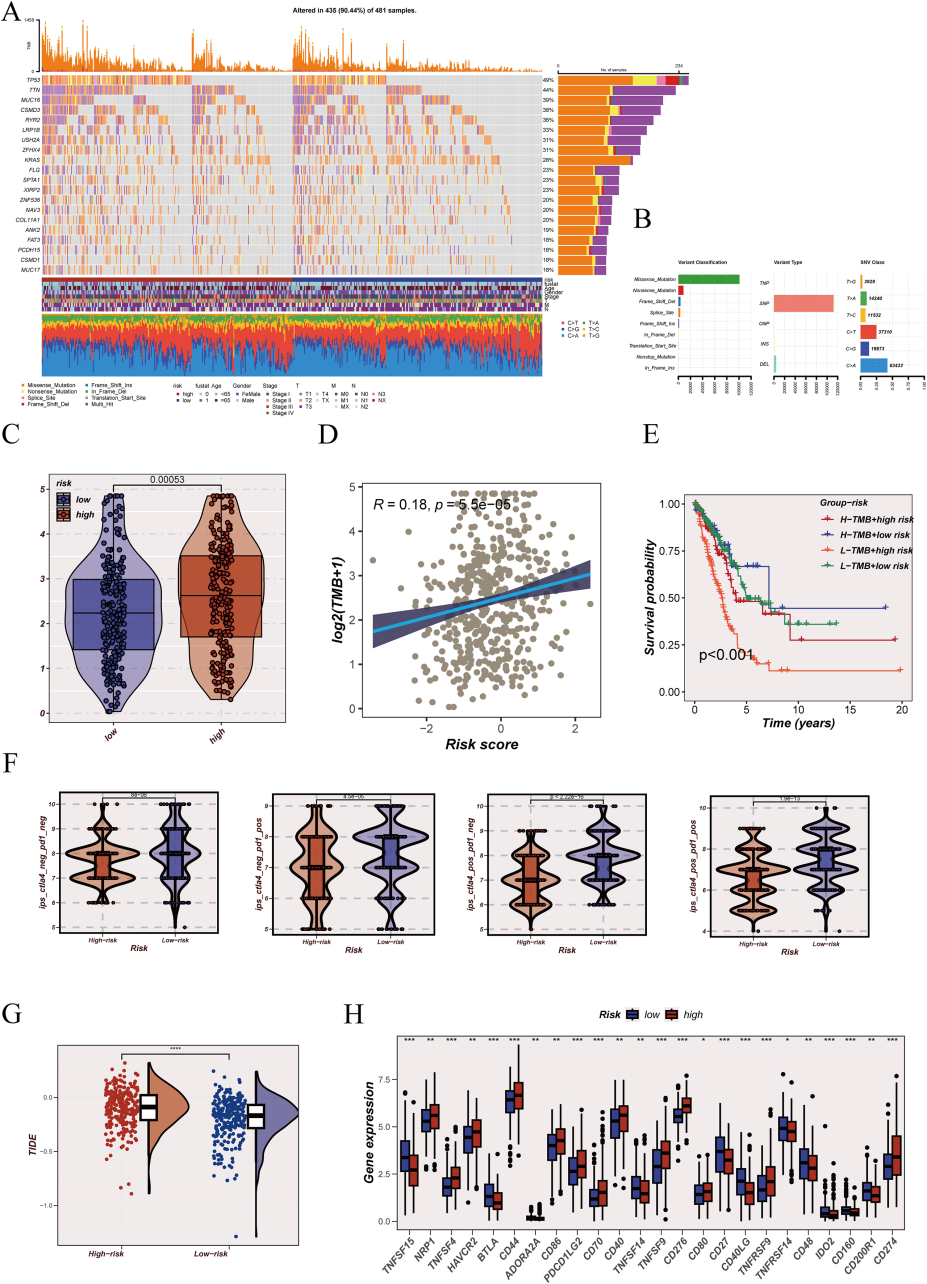
Genomic alteration patterns and immunotherapy-related indicators stratified by SAMRS score. **(A)** Oncoplot showing the somatic mutation landscape of TCGA-LUAD samples grouped by SAMRS score level with clinical annotations. **(B)** Summary panels of mutation-related features in TCGA-LUAD, including overall variant characteristics and distribution patterns. **(C)** Comparison of tumor mutational burden (TMB) between high- and low-SAMRS-score groups. **(D)** Association analysis between SAMRS score and TMB. **(E)** Kaplan–Meier survival analysis based on combined stratification of TMB status and SAMRS score group. **(F)** Immunophenoscore (IPS) comparison between high- and low-SAMRS-score groups. **(G)** TIDE score comparison between SAMRS score groups. **(H)** Differential expression of immune checkpoint–related genes between SAMRS score groups. *P < 0.05, **P < 0.01, ***P < 0.001, and P < 0.0001.

Somatic mutation profiling of the TCGA-LUAD cohort revealed a heterogeneous genomic alteration landscape across samples. When stratified by SAMRS score, mutation events were broadly distributed in both high- and low-SAMRS-score groups rather than being confined to a limited subset (**Figure 7A**). Mutation feature summary panels further outlined the overall variant composition and class-level mutation patterns of the cohort (**Figure 7B**). Tumor mutational burden showed a higher level in the high-SAMRS-score group compared with the low-SAMRS-score group (**Figure 7C**), and SAMRS score displayed a positive association with mutation load (**Figure 7D**), indicating that elevated model scores tend to coincide with increased genomic alteration intensity. Combined stratification using both TMB status and SAMRS score further separated patient survival patterns (**Figure 7E**). This joint grouping demonstrated clearer survival discrimination than either metric alone, supporting that SAMRS provides complementary prognostic information beyond mutation burden and refines outcome stratification within the same TMB category.

Immunotherapy-related response indicators showed consistent groupwise differences. IPS, which reflects predicted tumor immunogenicity and potential responsiveness to immune checkpoint blockade, was higher in the low-SAMRS-score group (**Figure 7F**), suggesting a more favorable immunotherapy response profile in this subgroup. In contrast, TIDE scores, which model immune evasion and treatment resistance likelihood, were elevated in the high-SAMRS-score group (**Figure 7G**), indicating a greater probability of immune escape and reduced checkpoint therapy benefit. Consistently, immune checkpoint–related genes displayed differential expression patterns between SAMRS groups (**Figure 7H**), supporting systematic variation in immune regulatory states across model strata.

Together, these results indicate that although the high-SAMRS-score group is characterized by higher mutation burden, the low-SAMRS-score group exhibits a more favorable predicted immunotherapy response profile, whereas the high-SAMRS-score group shows features associated with immune resistance.

### 3.8 Immune infiltration and immune functional variation across SAMRS score groups

To further characterize immune microenvironment differences associated with SAMRS, immune infiltration was evaluated using seven independent computational frameworks. Cross-method comparison showed consistent groupwise differences in multiple immune cell populations between high- and low-SAMRS-score groups (**Supplementary Figure 3A**), indicating that SAMRS-associated immune variation was reproducible across deconvolution strategies rather than method-specific.

Pathway-level GSVA analysis revealed systematic enrichment shifts between SAMRS groups (**Supplementary Figure 3B**). Immune activation, inflammatory signaling, and metabolic adaptation–related pathways were differentially represented across groups, suggesting coordinated remodeling of immune and stromal programs along the SAMRS gradient. Using ssGSEA-based immune cell scoring, multiple immune cell subsets showed distributional differences between SAMRS strata (**Supplementary Figure 3C**), supporting that the SAMRS score is associated with broad immune composition variation rather than isolated cell-type effects. Parallel ssGSEA functional signature analysis demonstrated that several immune-related functional modules, including immune activation and regulatory programs, varied between SAMRS groups (**Supplementary Figure 3D**), indicating coordinated differences in immune functional states.

### 3.9 Prognostic and functional features of TIMP1 across bulk and single-cell levels

Among the genes included in the SAMRS model signature, TIMP1 was selected for further focused analysis because it demonstrated consistent signal features across multiple analytical layers, including survival association, cross-dataset expression patterns, and immune-related network connectivity. Based on this stability and representativeness, TIMP1 was further characterized at bulk, single-cell, and regulatory network levels.

**Figure 8:**
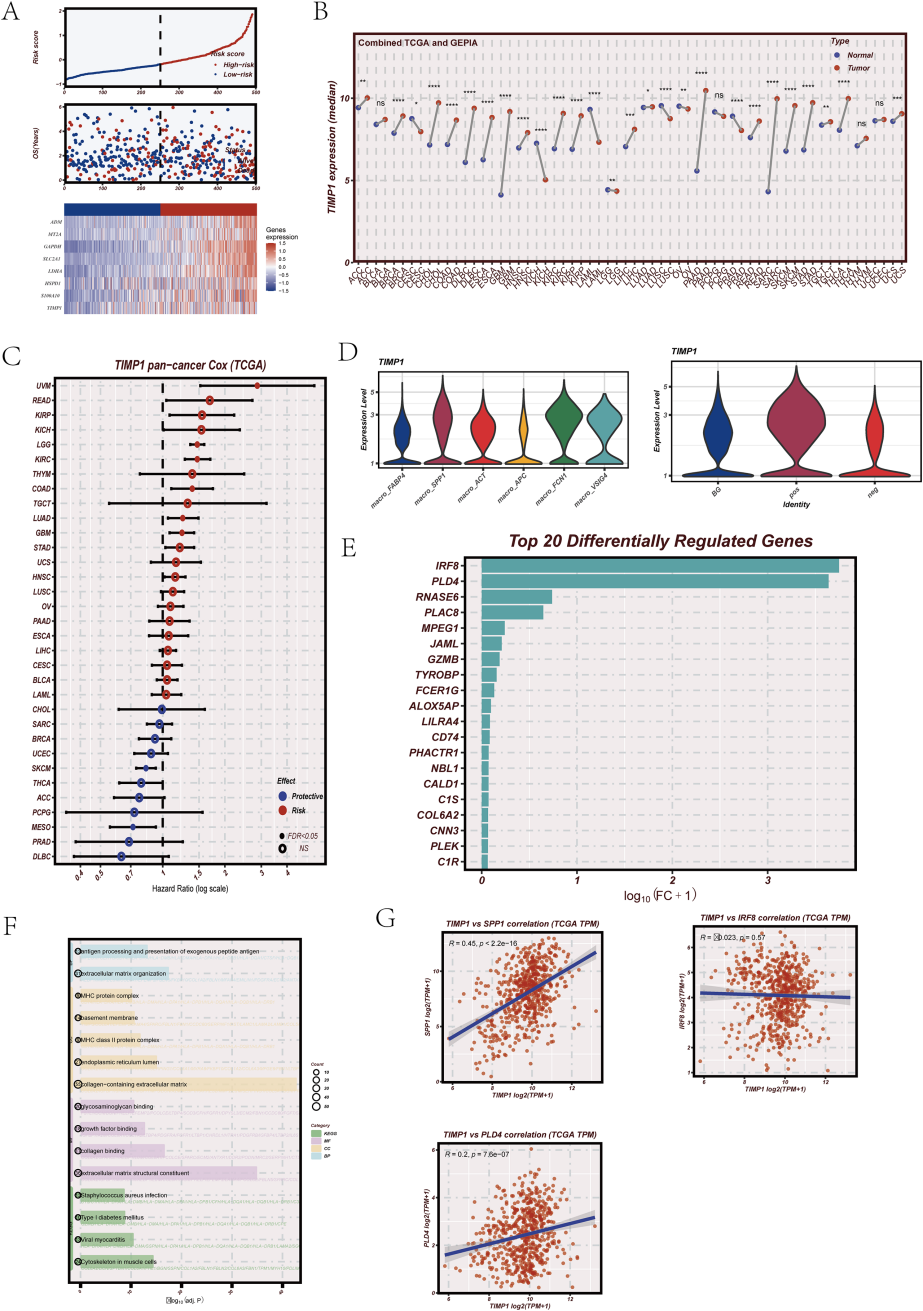
Model gene survival pattern, pan-cancer expression, and virtual knockout–associated functional effects centered on TIMP1. **(A)** Distribution of SAMRS model gene expression, risk score ranking, and survival status across the cohort. **(B)** TIMP1 expression across cancer types based on integrated TCGA tumor data and GTEx normal tissue data. **(C)** Pan-cancer univariate Cox regression analysis of TIMP1. **(D)** TIMP1 expression distribution across single-cell populations. **(E)** Top genes most strongly perturbed following TIMP1 virtual knockout in single-cell regulatory networks. **(F)** Functional enrichment analysis of genes showing the largest regulatory shifts after TIMP1 virtual knockout. **(G)** Correlation analysis between TIMP1 and representative immune-related genes including SPP1, IRF8, and PLD4.

When samples were ranked by SAMRS score, the combined visualization of model gene expression, risk score distribution, and survival status showed coordinated stratification patterns across the cohort (**Figure 8A**). Higher SAMRS score regions were enriched with adverse outcome events, while model gene expression displayed structured gradients along the ranking axis, supporting internal coherence between gene signature composition and risk assignment. To obtain a more stable normal reference baseline, tumor expression data from TCGA were analyzed together with normal tissue data from GTEx, given the limited number of normal samples available within TCGA alone. Under this integrated framework, TIMP1 expression was found to be broadly elevated across multiple tumor types relative to normal tissues (**Figure 8B**), indicating a cross-cancer upregulation pattern rather than a dataset-specific shift. Pan-cancer survival screening further showed that TIMP1 demonstrated prognostic associations across several malignancies (**Figure 8C**), supporting broader clinical relevance beyond a single tumor type. At the single-cell level, TIMP1 expression showed preferential enrichment in specific cellular compartments rather than uniform distribution across all cell types (**Figure 8D**), suggesting cell-context–dependent functional involvement within the tumor microenvironment. Single-cell regulatory network–based virtual knockout analysis identified a defined set of genes most strongly perturbed after TIMP1 removal (**Figure 8E**). Genes with the largest regulatory shifts were mainly enriched in immune regulation, inflammatory response, and stress-adaptation pathways (**Figure 8F**), indicating that TIMP1-centered regulatory structure is functionally connected to immune-associated programs. Correlation analysis demonstrated coordinated expression behavior between TIMP1 and immune-related genes including SPP1, IRF8, and PLD4 (**Figure 8G**), supporting the presence of a shared macrophage-linked regulatory module.

### 3.10 Spatial co-localization of TIMP1 and macrophage-associated signals

**Figure 9:**
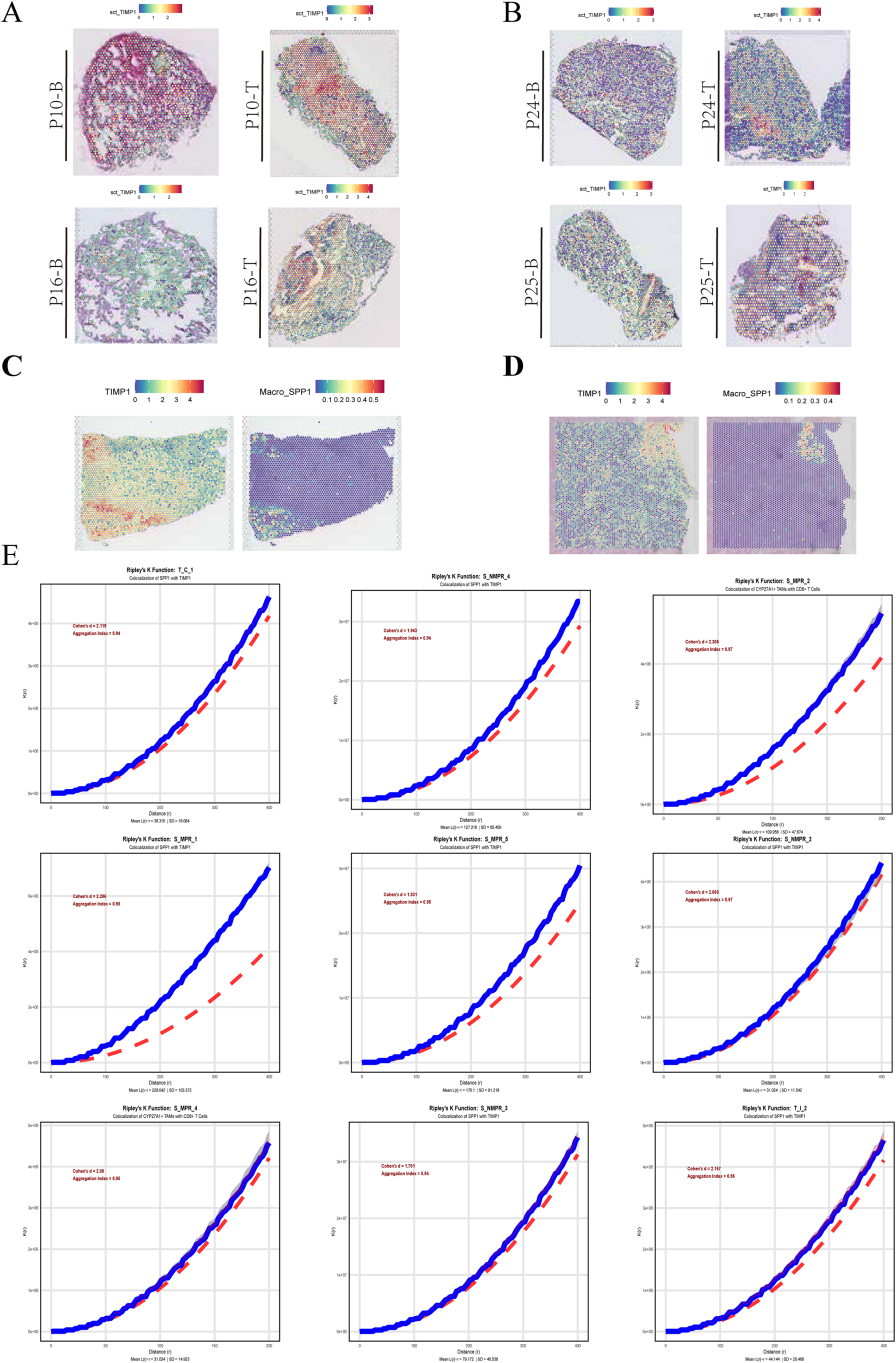
Spatial transcriptomic distribution and spatial association analysis of TIMP1 and macrophage-related signals. **(A, B)** Spatial transcriptomic maps showing gene expression intensity patterns across multiple tumor sections. **(C, D)** Spatial feature plots of TIMP1 and macrophage-associated signatures across representative tissue regions. **(E)** Spatial co-localization analysis between TIMP1-positive and macrophage-associated spots using spatial clustering statistics across multiple samples.

Spatial transcriptomic profiling revealed heterogeneous gene expression patterns across tumor tissue sections, with clear regional variation in signal intensity (**Figures 9A, B**). These spatial maps indicated that transcriptional activity was not uniformly distributed but instead formed structured expression domains within the tumor microenvironment. Spatial feature visualization further showed that TIMP1 expression and macrophage-associated signatures exhibited regionally enriched patterns rather than diffuse distribution (**Figures 9C, D**). High-intensity TIMP1 signals frequently appeared in spatially restricted zones that overlapped with macrophage-related expression features, suggesting localized co-enrichment.Spatial association analysis based on clustering statistics demonstrated that TIMP1-positive regions and macrophage-associated spots showed non-random spatial proximity across multiple sections (**Figure 9E**). The observed aggregation patterns exceeded background spatial randomness, supporting the presence of structured spatial co-localization between TIMP1 activity and macrophage-related niches.

Together, these spatial analyses provide tissue-level evidence that TIMP1-associated signals are spatially coupled with macrophage-enriched regions within the tumor microenvironment.

### 3.11 TIMP1 silencing inhibits tumor cell proliferation and migration *in vitro* and *in vivo*

**Figure 10:**
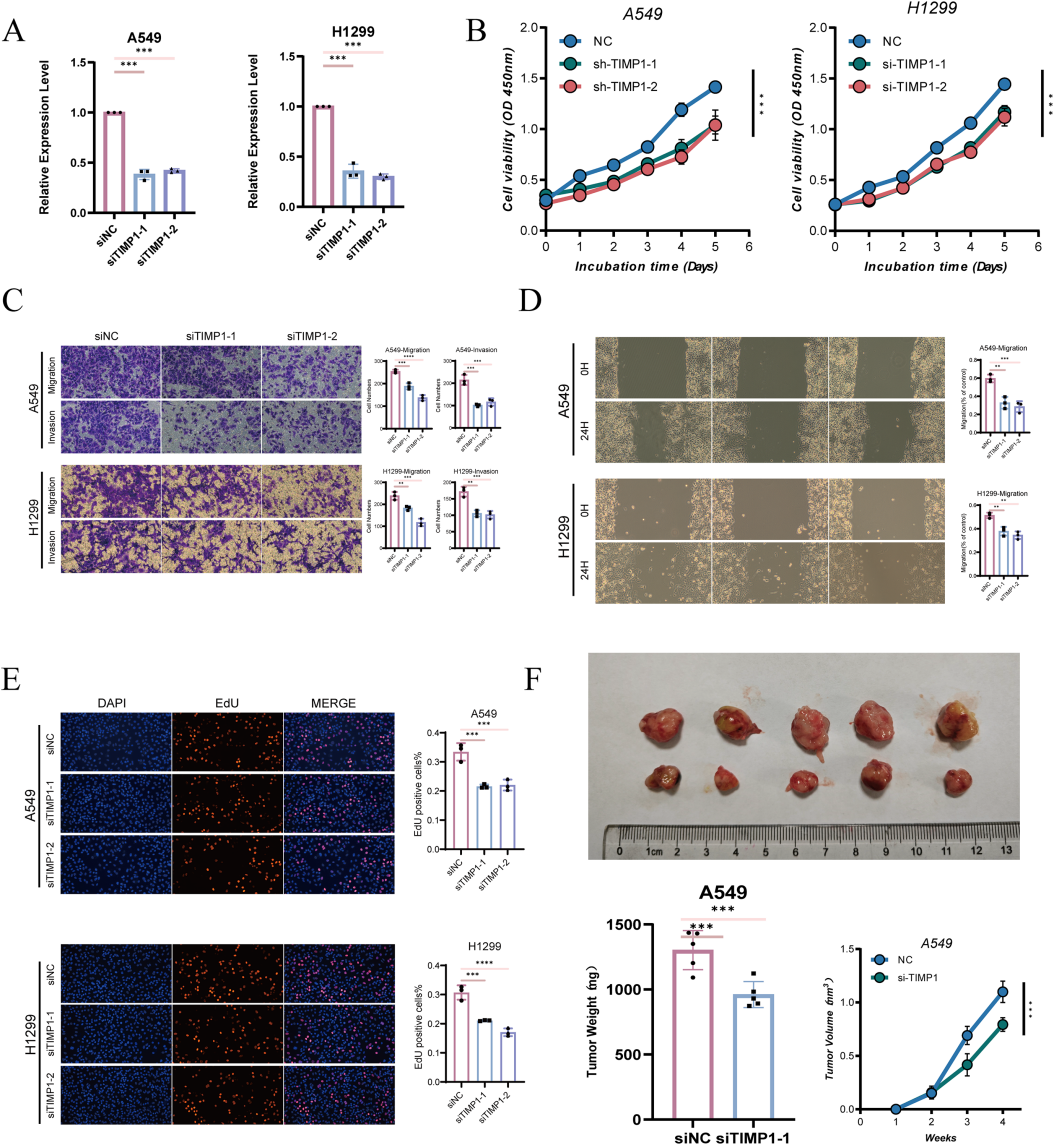
Functional validation of TIMP1 knockdown in lung cancer cell models. **(A)** Knockdown efficiency of TIMP1 in A549 and H1299 cells. **(B)** Cell viability curves following TIMP1 silencing. **(C)** Transwell migration and invasion assays after TIMP1 knockdown. **(D)** Wound-healing assays evaluating migratory capacity after TIMP1 suppression. **(E)** EdU incorporation assays measuring proliferative activity under TIMP1 knockdown. **(F)** Xenograft tumor growth comparison between control and TIMP1-silenced groups, including tumor size, weight, and growth curves. **P < 0.01, ***P < 0.001, and ****P < 0.0001.

To functionally validate the role of TIMP1, loss-of-function experiments were performed in lung cancer cell lines. TIMP1 expression was effectively reduced in both A549 and H1299 cells following siRNA-mediated silencing (**Figure 10A**), confirming successful knockdown at the transcript level. Cell viability assays showed that TIMP1 suppression led to reduced growth capacity over time in both cell models (**Figure 10B**), indicating that TIMP1 contributes to proliferative maintenance. Transwell assays demonstrated that TIMP1 knockdown markedly decreased both migratory and invasive cell fractions (**Figure 10C**). Consistently, wound-healing experiments showed slower closure dynamics under TIMP1 silencing conditions (**Figure 10D**), supporting a reduction in motility-related behavior. EdU incorporation assays further revealed decreased proliferative activity after TIMP1 inhibition (**Figure 10E**), providing additional evidence that TIMP1 supports cell-cycle–associated growth programs. *In vivo* xenograft experiments showed that tumors derived from TIMP1-silenced cells displayed reduced growth compared with controls, with smaller tumor size and lower tumor weight observed at endpoint measurement (**Figure 10F**). Tumor growth curves also indicated sustained suppression of tumor expansion following TIMP1 knockdown.

Together, these *in vitro* and *in vivo* findings support a functional role for TIMP1 in promoting tumor cell proliferation, migration, invasion, and tumor growth.

## 4 Discussion

LUAD remains a major contributor to cancer mortality despite continuous progress in targeted therapy and immunotherapy (29). Increasing evidence indicates that tumor behavior is not solely determined by malignant epithelial programs but is tightly shaped by the immune microenvironment (30). Among immune populations, macrophages represent a dominant and highly plastic component that can adopt diverse functional states under tumor-driven signaling pressure (31). This functional diversity has been linked to immune suppression, extracellular matrix remodeling, and therapy resistance, yet translating macrophage heterogeneity into clinically actionable stratification tools has remained challenging (32).

Single-cell profiling has substantially expanded the understanding of macrophage diversity in lung cancer (33, 34). In particular, SPP1-positive macrophages have repeatedly been associated with pro-tumorigenic and tissue-remodeling phenotypes, often enriched in advanced or invasive tumor contexts (35). The present analysis similarly identified SPP1-enriched macrophage subsets with pathway features consistent with metabolic activation and tumor-supportive programs (36, 37). These observations support the concept that not all tumor-associated macrophages are functionally equivalent and that specific transcriptional states may carry stronger prognostic and biological relevance (38, 39). Taken together, our data suggest that the identified macrophage subsets are more likely to represent interconnected functional states rather than completely independent populations. The combination of subset-specific enrichment patterns, Scissor-based phenotype mapping, trajectory inference, and cell–cell communication analysis indicates that distinct macrophage programs may coexist and transition within the LUAD microenvironment, with some states appearing more inflammatory or immune-reactive, and others more closely associated with tissue remodeling, metabolic adaptation, and tumor-supportive activity. This interpretation provides a broader biological framework for understanding how macrophage heterogeneity may contribute to prognosis and microenvironmental regulation in LUAD.

A key challenge in single-cell studies is linking transcriptional cell states directly to patient-level outcomes. The Scissor framework provides a phenotype-guided mapping strategy that connects bulk clinical signals to individual single cells, enabling the identification of prognosis-associated cellular subsets rather than relying solely on unsupervised clustering (40). Applying this strategy highlighted Scissor+ macrophage populations associated with adverse outcomes and oncogenic pathway coupling. This phenotype-linked cell selection step provides a biologically grounded basis for downstream model construction and reduces the risk of purely statistical feature selection disconnected from cellular context.

Building on Scissor-derived macrophage signals, a prognostic model termed the SAMRS was developed and validated across cohorts. The model demonstrated stable survival stratification ability and showed structured associations with genomic and immune-related features. Notably, higher SAMRS scores were associated with increased mutation burden but also with stronger immune-evasion signatures, whereas lower SAMRS scores aligned with more favorable predicted immunotherapy response profiles. This pattern suggests that mutation load alone may not fully capture immune responsiveness and that macrophage-associated transcriptional programs provide complementary stratification information.

Cell–cell communication analysis further positioned macrophages as central nodes within intercellular signaling networks, with FN1-related interaction axes emerging as prominent pathways. FN1 is known to participate in extracellular matrix organization, adhesion signaling, and stromal–immune crosstalk. Its centrality in macrophage-associated communication networks supports a model in which macrophages contribute to tumor progression not only through soluble immune mediators but also through matrix-linked signaling structures that reshape the tumor niche (41, 42).

Among model-associated genes, TIMP1 showed consistent signals across bulk transcriptomics, single-cell distribution, regulatory network perturbation, and spatial transcriptomics. Network-based virtual knockout suggested broad downstream regulatory effects linked to immune and inflammatory programs, while spatial analysis indicated regional co-enrichment with macrophage-associated signals. Functional experiments further demonstrated that TIMP1 suppression reduced proliferative and migratory capacity and restrained tumor growth *in vivo*. Together, these layers of evidence support TIMP1 as a macrophage-associated regulatory node connected to tumor-promoting programs rather than a passive marker.

Several strengths characterize this study, including phenotype-guided single-cell mapping, multi-cohort validation of a macrophage-derived risk score, and multi-layer validation integrating communication inference, regulatory perturbation, spatial transcriptomics, and functional experiments. Limitations should also be noted. The computational framework relies on retrospective public datasets, and prospective clinical validation is still required. In addition, although functional assays support TIMP1 involvement, the upstream regulatory triggers and downstream pathway dependencies warrant deeper mechanistic investigation.

Overall, this work links macrophage phenotype heterogeneity with prognostic modeling and functional validation in LUAD. The SAMRS framework and TIMP1-centered regulatory signals provide a basis for further exploration of macrophage-guided risk stratification and therapeutic targeting strategies.

## Data Availability

The original contributions presented in the study are included in the article/**Supplementary Material**. Further inquiries can be directed to the corresponding authors.

## Supplementary material

Supplementary Figure 1 Survival stratification and time-dependent ROC performance based on median model risk score. **(A)** Kaplan–Meier survival curves comparing high- and low-risk groups defined by the median model risk score across the TCGA cohort and multiple independent validation cohorts. **(B)** Time-dependent ROC curves evaluating 1-, 3-, and 5-year survival prediction accuracy of the model across discovery and validation datasets.

Supplementary Figure 2 Performance comparison between SAMRS and previously reported LUAD prognostic models in multiple cohorts. Forest-style comparison of the C-index for SAMRS and 20 previously published LUAD prognostic models across the TCGA cohort and six independent GEO validation cohorts (GSE13213, GSE26939, GSE30219, GSE31210, GSE50081, and GSE42127). Dots indicate model-specific C-index values, and horizontal lines indicate confidence intervals. Previously reported models are identified by PubMed ID.

Supplementary Figure 3 Immune infiltration and immune functional differences between SAMRS score groups. **(A)** Immune cell infiltration estimates across seven computational methods comparing high- and low-SAMRS-score groups. **(B)** GSVA enrichment analysis showing pathway-level differences between SAMRS score groups. **(C)** ssGSEA-based comparison of immune cell population scores between SAMRS score groups. **(D)** ssGSEA-based comparison of immune-related functional signature scores between SAMRS score groups.
