# Supplementary figures and images for: A scissor-guided single-cell framework defines a macrophage-derived risk score for prognostic and immunotherapy stratification in lung adenocarcinoma
Source: Front Immunol. 2026 May 14;17:1827555. doi: 10.3389/fimmu.2026.1827555 (PMC13215902; doi:10.3389/fimmu.2026.1827555)

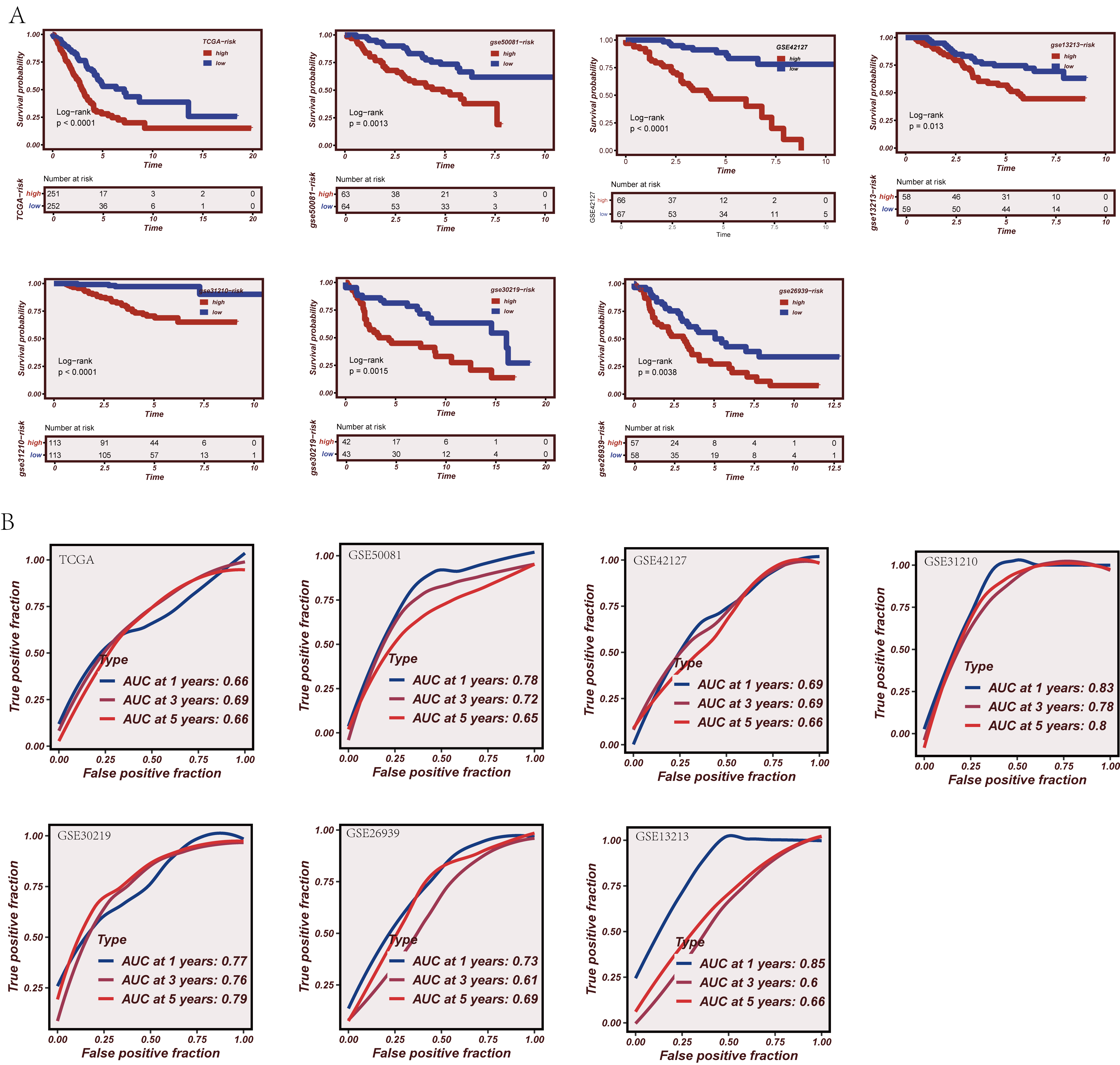

Supplement: Supplementary Figure 1 — Survival stratification and time-dependent ROC performance based on median model risk score. (A) Kaplan–Meier survival curves comparing high- and low-risk groups defined by the median model risk score across the TCGA cohort and multiple independent validation cohorts. (B) Time-dependent ROC curves evaluating 1-, 3-, and 5-year survival prediction accuracy of the model across discovery and validation datasets. [file Image1.tif]

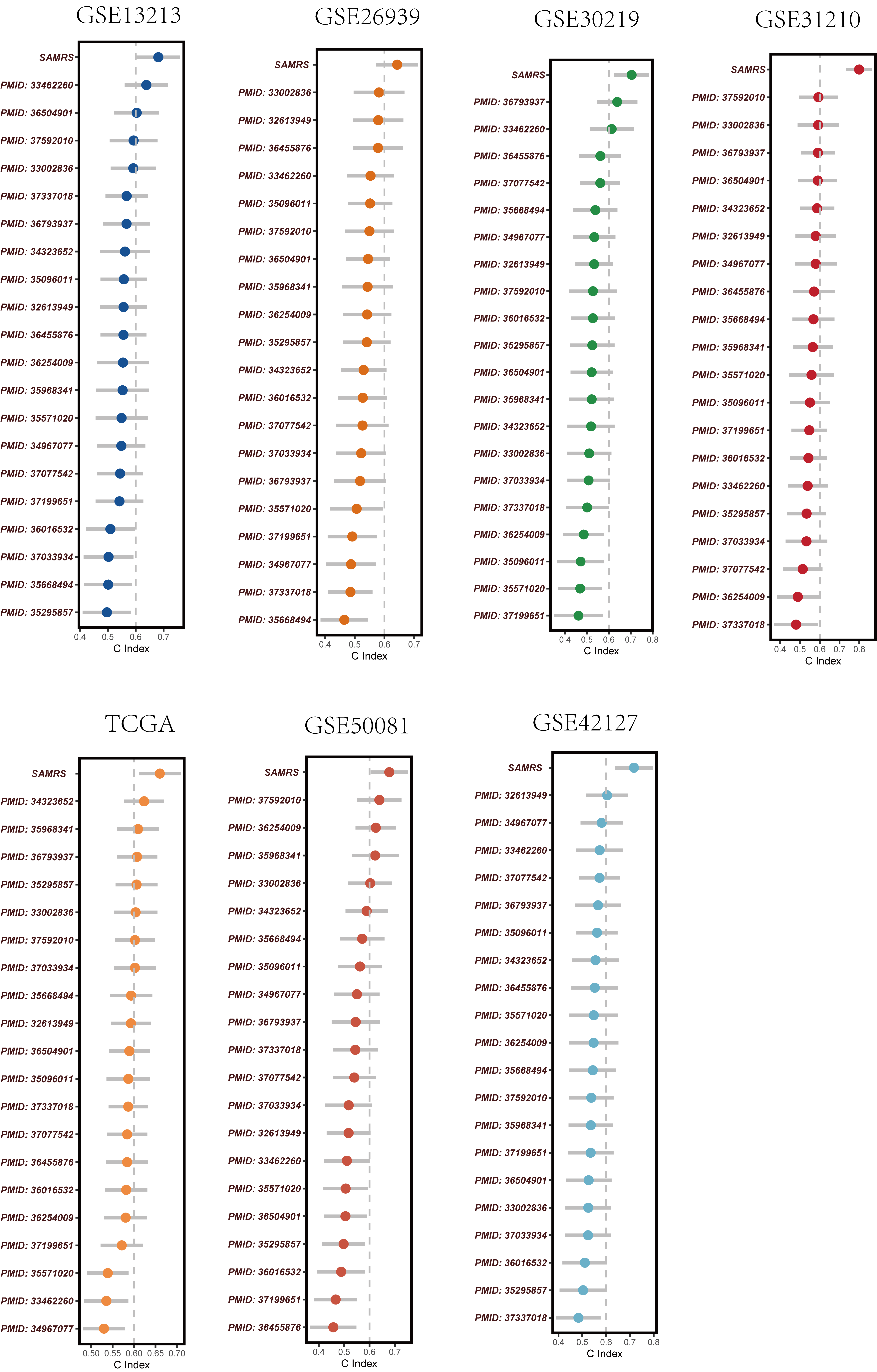

Supplement: Supplementary Figure 2 — Performance comparison between SAMRS and previously reported LUAD prognostic models in multiple cohorts. Forest-style comparison of the C-index for SAMRS and 20 previously published LUAD prognostic models across the TCGA cohort and six independent GEO validation cohorts (GSE13213, GSE26939, GSE30219, GSE31210, GSE50081, and GSE42127). Dots indicate model-specific C-index values, and horizontal lines indicate confidence intervals. Previously reported models are identified by PubMed ID. [file Image2.tif]

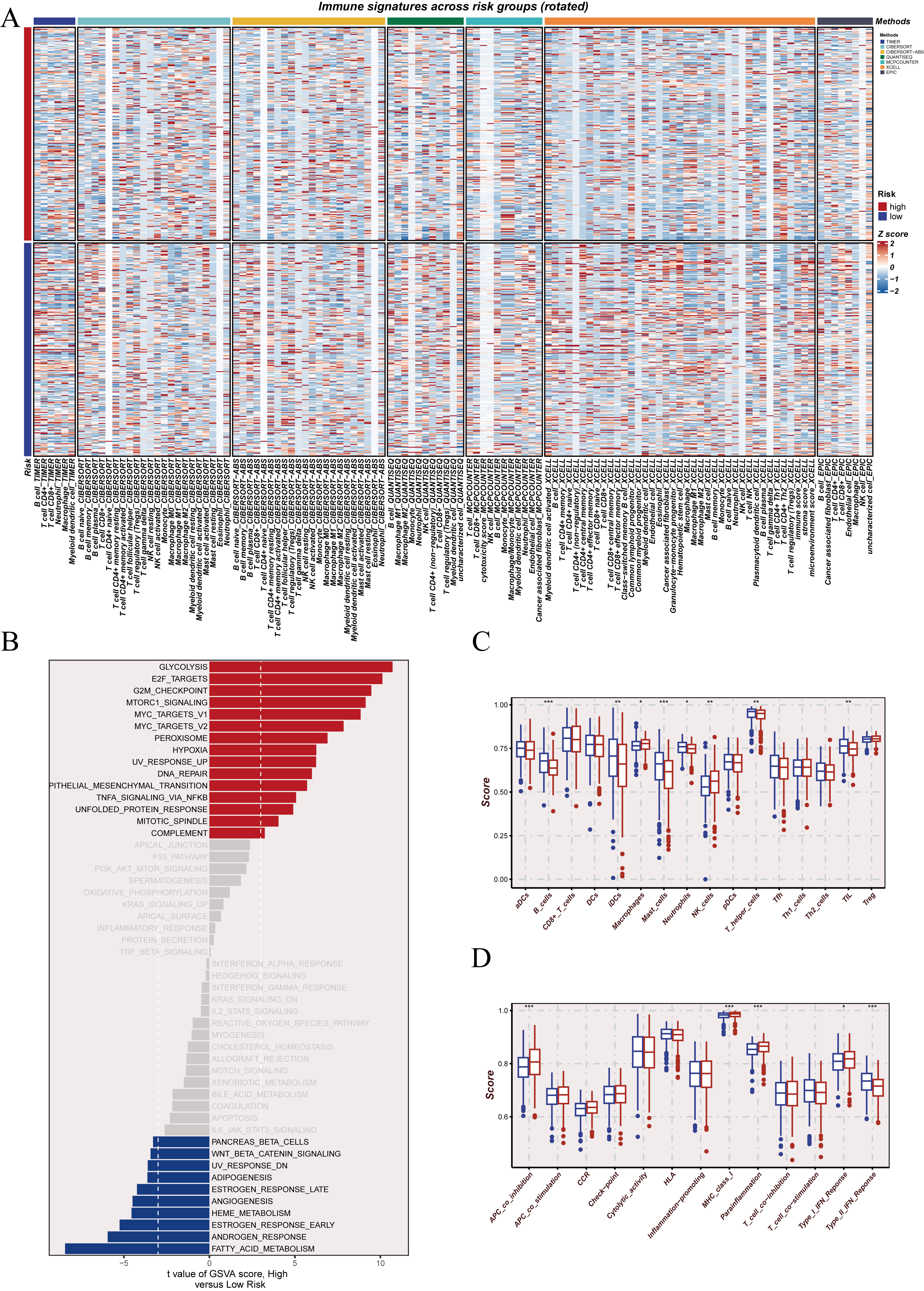

Supplement: Supplementary Figure 3 — Immune infiltration and immune functional differences between SAMRS score groups. (A) Immune cell infiltration estimates across seven computational methods comparing high- and low-SAMRS-score groups. (B) GSVA enrichment analysis showing pathway-level differences between SAMRS score groups. (C) ssGSEA-based comparison of immune cell population scores between SAMRS score groups. (D) ssGSEA-based comparison of immune-related functional signature scores between SAMRS score groups. [file Image3.tif]
